# Endothelial Cell Metabolism in Atherosclerosis

**DOI:** 10.3389/fcell.2018.00082

**Published:** 2018-08-07

**Authors:** Kosta Theodorou, Reinier A. Boon

**Affiliations:** ^1^Centre of Molecular Medicine, Institute of Cardiovascular Regeneration, Goethe-University, Frankfurt am Main, Germany; ^2^German Center for Cardiovascular Research DZHK, Partner Site Rhine-Main, Berlin, Germany; ^3^Department of Physiology, Amsterdam Cardiovascular Sciences, VU University Medical Center, Amsterdam, Netherlands

**Keywords:** endothelial cells, metabolism, atherosclerosis, inflammation, hyperglycemia, hyperlipidemia, aging

## Abstract

Atherosclerosis and its sequelae, such as myocardial infarction and stroke, are the leading cause of death worldwide. Vascular endothelial cells (EC) play a critical role in vascular homeostasis and disease. Atherosclerosis as well as its independent risk factors including diabetes, obesity, and aging, are hallmarked by endothelial activation and dysfunction. Metabolic pathways have emerged as key regulators of many EC functions, including angiogenesis, inflammation, and barrier function, processes which are deregulated during atherogenesis. In this review, we highlight the role of glucose, fatty acid, and amino acid metabolism in EC functions during physiological and pathological states, specifically atherosclerosis, diabetes, obesity and aging.

## Introduction

Cardiovascular diseases (CVDs) are the leading cause of morbidity and mortality worldwide (World Health Organization, 2014). The underlying pathology of the majority of cardiovascular-related deaths is atherosclerosis, which is a lipid-driven chronic inflammatory disease of the medium- and large-sized arteries (Lusis, 2000). One of the earliest detectable changes in the development of atherosclerosis is endothelial cell (EC) activation and dysfunction at lesion-prone areas of the arterial vasculature (Hajra et al., 2000). These areas, particularly at bends and branch points, are characterized by ECs that display a pro-inflammatory, pro-thrombotic phenotype with a reduced barrier function, which is prompted by disturbed blood flow dynamics (Tabas et al., 2015). Moreover, subendothelial retention and modification of apolipoprotein B-containing lipoproteins, such as low-density lipoproteins (LDL), further activate ECs. This triggers an inflammatory response, leading to the recruitment of monocytes into the intima where they differentiate into macrophages and engulf modified lipoproteins to become foam cells. Progressed atherosclerotic lesions are characterized by a fibrous cap overlaying a lipid rich, necrotic core, and an accumulation of leukocytes in the lateral edges, which promote plaque instability by modulating EC phenotype and through proteolytic degradation of extracellular matrix (ECM) components. Unstable lesions may, upon rupture, lead to an atherothrombotic event, causing clinical events such as myocardial infarction or stroke. Alternatively, superficial intimal erosions, as a consequence of EC apoptosis and detachment, may also trigger an atherothrombotic event and its clinical sequelae (Quillard et al., 2015; Tabas et al., 2015).

Although initially believed to be an inert, semi-permeable barrier between blood constituents, and the underlying subendothelial tissues, the endothelium is now viewed as a metabolically active organ that plays a crucial role in vascular homeostasis and throughout the life history of atherosclerosis. In this review, we will focus on EC metabolism in health and atherosclerosis development. First, we will briefly review the three major metabolic pathways [i.e., glucose, fatty acids (FA) and amino acids (AA)] in ECs, followed by the role of EC metabolism in atherosclerosis.

## Endothelial cell metabolism in health

To date, studies investigating EC metabolism have been limited to using cultured ECs, whose metabolism may be rewired because of *in vitro* conditions that do not fully recapitulate the *in vivo* environment (Hensley et al., 2016; Cantor et al., 2017). Nevertheless, great strides have been made in dissecting the roles of metabolism in EC functions by conditional targeting of key metabolic genes *in vivo*.

### Glucose metabolism

Glycolysis is the main energy supplier in cultured ECs, accounting for ~75–85% of the total ATP production (Krützfeldt et al., 1990; De Bock et al., 2013). It has been estimated that isolated coronary microvascular ECs metabolize ~99% of glucose into lactate under aerobic conditions, while only 0.04% is oxidized in the tricarboxylic acid (TCA) cycle (Krützfeldt et al., 1990). Indeed, inhibiting glycolysis using 2-deoxy-D-glucose induces EC cytotoxicity, indicating that glucose is essential for proper EC functioning and maintenance (Merchan et al., 2010).

Upon stimulation with pro-angiogenic signals, like vascular endothelial growth factor (VEGF) and fibroblast growth factor 2, ECs increase their glycolytic flux to support proliferation and migration (De Bock et al., 2013; Yu et al., 2017). In contrast, limiting glycolysis by pharmacological inhibition or genetic silencing of phosphofructokinase-2/fructose-2,6-biphosphatase 3 (PFKFB3) or silencing of hexokinase (HK)2 impairs EC proliferation, migration and vascular sprouting (De Bock et al., 2013; Schoors et al., 2014; Yu et al., 2017). Moreover, endothelial-specific PFKFB3 or HK2 deficiency causes vascular hypobranching in mice (De Bock et al., 2013; Yu et al., 2017).

**Figure 1:**
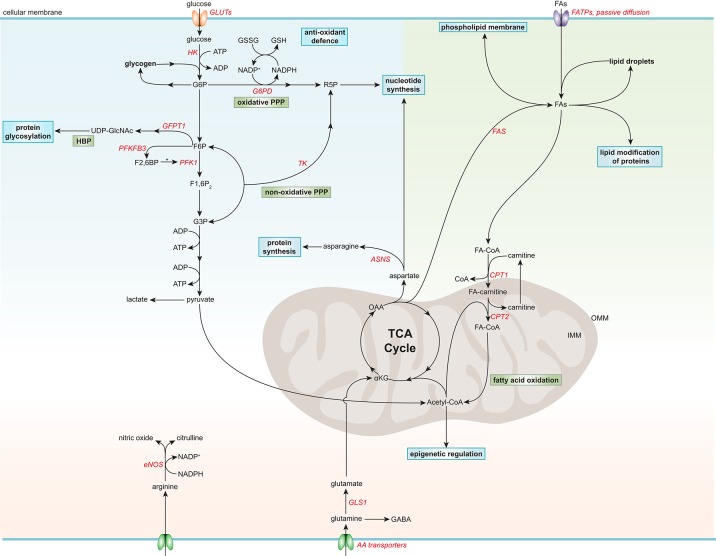
Simplified general overview of glucose, fatty acid, and amino acid metabolism in healthy endothelial cells. Glucose enters ECs via glucose transporters (GLUTs), which is converted to glucose-6-phosphate (G6P) by hexokinase (HK) at the expense of adenosine triphosphate (ATP). G6P can be converted to glycogen for storage or used in the oxidative pentose phosphate pathway (PPP). The oxidative PPP generates reduced glutathione (GSH) and ribose-5-phosphate (R5P), which are used in the anti-oxidant defense and nucleotide synthesis, respectively. G6P can be further metabolized to fructose-6-phosphate (F6P) which is converted to uridine diphosphate N-acetylglucosamine (UDP-GlcNAc), a substrate used for protein glycosylation, in the hexosamine biosynthesis pathway (HBP). F6P can also be converted to fructose-2,6-bisphophate (F2,6BP) by 6-phosphofructo-2-kinase/fructose-2,6-bisphosphatase 3 (PFKF3B), the main isoform in ECs. F2,6BP is a positive allosteric regulator of phosphofructokinase 1 (PFK1), which converts F6P to fructose-1,6-bisphophate (F1,6P_2_), which is further metabolized to glyceraldehyde-3-phosphate (G3P). G3P together with F6P can be used in the non-oxidative PPP by transketolase (TK) to eventually produce R5P. G3P can also undergo several conversion steps leading to the production of ATP from adenosine diphosphate (ADP) and pyruvate, which can be further metabolized to lactate. After being taken up by fatty acid (FA) transporters (FATPs) or through passive diffusion, FAs become metabolically activated by coupling to coenzyme A (CoA). For FA oxidation (FAO) to occur, FAs have to be imported into the mitochondria, which is carried out by the acyl-carnitine shuttle system. At the outer mitochondrial membrane (OMM), carnitine palmitoyltransferase 1 (CPT1) converts FA-CoAs into FA-carnitines which facilitates the transport of FAs across the inner mitochondrial membrane (IMM) where they are converted back to FA-CoA by CPT2 (located in the IMM). FAO generates ATP and acetyl-CoA which is used in the tricarboxylic acid cycle (TCA). TCA intermediates are used for nucleotide synthesis or FA production, facilitated by fatty acid synthase (FAS). FAs can be stored in lipid droplets, converted to phospholipids to maintain the cell membrane or used for lipid modification of proteins. The amino acid (AA) glutamine is the most consumed AA in ECs. Once inside the cell, glutamine is converted to glutamate by glutaminase 1 (GLS1), which is further metabolized to α-ketoglutarate (αKG), a key intermediate in the TCA cycle. Through several metabolic steps, αKG is converted to oxaloacetic acid (OAA), which can be used for the generation of the AA aspartate. Aspartate can be used as a precursor for nucleotide synthesis or converted to the AA asparagine by asparagine synthetase (ASNS) using an ammonia group from glutamine thereby generating glutamate. Another important AA in ECs is arginine, which is used for the generation of the anti-atherogenic gaseous molecule nitric oxide using nicotinamide adenine dinucleotide phosphate (NADPH) as a cofactor. G6PD, glucose-6-phosphate dehydrogenase; GFPT1, glutamine:fructose-6-phosphate amidotransferase 1; GSSG, oxidized glutathione; NADP^+^, nicotinamide adenine dinucleotide.

Apart from using glucose for energy production, there are also alternative fates for glucose in ECs (Lunt and Vander Heiden, 2011; Figure 1). After glucose is taken up by ECs through facilitated diffusion by the glucose transporters (GLUT), primarily by GLUT1, glucose is phosphorylated by HK to glucose-6-phosphate (G6P) (Mann et al., 2003; Lunt and Vander Heiden, 2011). G6P can be converted to and stored as glycogen or processed in the oxidative branch of the pentose phosphate pathway (oxPPP) to yield nicotinamide adenine dinucleotide phosphate (NADPH) and ribose-5-phosphate (R5P), which are used for antioxidant defense and nucleotide biosynthesis, respectively (Lunt and Vander Heiden, 2011; Adeva-Andany et al., 2016). Silencing of the G6P dehydrogenase (G6PD), the rate-limiting enzymes of oxPPP, reduces EC proliferation and migration, and increases cellular reactive oxygen species (ROS) (Leopold et al., 2003). Furthermore, ECs contain glycogen reservoirs and pharmacological inhibition of glycogen phosphorylase, the rate-limiting enzyme in glycogen degradation, impairs EC migration and viability (Vizán et al., 2009). However, how glycogen metabolism affects EC functions remains to be determined.

G6P can also be further metabolized to fructose-6-phosphate (F6P), which can be shunted into the hexosamine biosynthesis pathway (HBP) to produce UDP-N-acetylglucosamine (UDP-GlcNAc), an important substrate for protein O-linked glycosylation that is critical for a plethora of EC functions (Laczy et al., 2009). Silencing of the rate-limiting enzyme in the HBP, glutamine:fructose-6-phosphate amidotransferase 1, augments VEGF-induced vascular sprouting (Zibrova et al., 2017). Furthermore, the two glycolysis intermediates F6P and glyceraldehyde-3-phosphate (G3P) can be used in the non-oxidative branch of the pentose phosphate pathway (non-oxPPP) to generate R5P, but not NADPH, in contrast to oxPPP (Lunt and Vander Heiden, 2011). Pharmacological inhibition of transketolase, the rate-limiting enzyme in non-oxPPP, limits ECs viability, and migration (Vizán et al., 2009).

ECs have a relatively low mitochondrial content (<2–12% of the total cellular volume) compared to other cell types, such as hepatocytes (28%) and cardiomyocytes (22–37%, depending on the species; Blouin et al., 1977; Oldendorf et al., 1977; Barth et al., 1992). This is also reflected by the lower mitochondrial respiration of ECs compared to more oxidative cell types, like hepatocytes and cardiomyocytes (De Bock et al., 2013). Nevertheless, ECs have a considerable spare mitochondrial respiratory capacity (Doddaballapur et al., 2015; Wilhelm et al., 2016), which may be called upon during stress conditions like glucose deprivation to metabolize alternative substrates, such as FAs and glutamine (Krützfeldt et al., 1990; Mertens et al., 1990), however this remains to be determined. Cultured ECs derive only ~15% of the total amount of ATP via oxidative pathways, suggesting that rather by playing a major role in energy production, mitochondria in ECs are more likely to have a signaling function (by producing ROS and maintaining intracellular Ca^2+^ homeostasis) and support biomass synthesis (by generating metabolic intermediates; Quintero et al., 2006; De Bock et al., 2013; Tang et al., 2014; Schoors et al., 2015; Huang et al., 2017).

### Fatty acid metabolism

FAs in cultured ECs act as a carbon source for the production of the AA aspartate (a nucleotide precursor) and deoxyribonucleotides which are required for DNA synthesis, rather than supplying energy (accounting for <5% of the total amount of ATP produced) or maintaining redox homeostasis (Figure 1; Schoors et al., 2015). Endothelial-specific deficiency or silencing of carnitine palmitoyltransferase (CPT)1A, the rate-limiting enzyme in FA oxidation (FAO), causes vascular sprouting defects *in vivo* and *in vitro* owing to a reduction in proliferation, but not migration (Schoors et al., 2015). In addition, pharmacological inhibition of CPT1 or silencing CPT1A or CPT2 reduces FAO and enhances endothelial permeability (Patella et al., 2015; Xiong et al., 2018). Furthermore, during lymphatic EC (LEC) differentiation, LECs upregulate CPT1A to support their proliferation, but also to promote their differentiation through acetyl-coenzyme A (acetyl-CoA) production, which is used for histone acetylation of lymphatic genes (Wong et al., 2017). Moreover, FAO maintains the cellular pool of acetyl-CoA and retains the identity of vascular ECs by reducing transforming growth factor β-induced endothelial-to-mesenchymal transition (EndMT) (Xiong et al., 2018). In addition to its role in EC proliferation, differentiation and permeability, FA metabolism also modulates the lipid composition of EC membranes, thereby regulating membrane stiffness and multiple cellular functions (Caires et al., 2017; Glatzel et al., 2018; Harayama and Riezman, 2018).

Besides using FAs for their own needs, ECs regulate the transport of FAs toward metabolically active tissues, such as skeletal and cardiac muscle (Mehrotra et al., 2014). Circulating FAs, either bound to albumin or locally released from triglyceride-rich lipoproteins through lipoprotein lipase-mediated lipolysis at the luminal surface of ECs, can enter ECs via passive diffusion or by FA transporter proteins. Interestingly, ECs readily store FAs in lipid droplets as a protective measure against endoplasmic reticulum (ER) stress (Kuo et al., 2017). Furthermore, FAs can be liberated from lipid droplets, which can be used by the ECs themselves or released to the underlying tissues (Kuo et al., 2017).

Although there is a continuous supply of FA from the blood stream that can enter cells, ECs also have the capability to synthesize FA *de novo*, since they express FA synthase (FAS; Wei et al., 2011; Hagberg et al., 2013). Silencing or genetic deletion of FAS impairs EC migration, vascular sprouting and permeability, and proper endothelial nitric oxide synthase (eNOS) functioning by reducing its palmitoylation (Wei et al., 2011).

### Amino acid metabolism

Glutamine is the most consumed AA in ECs and is crucial for angiogenesis both *in vitro* and *in vivo* by contributing to TCA cycle anaplerosis, biomass synthesis and redox homeostasis (Huang et al., 2017; Kim B. et al., 2017). Withdrawal of glutamine, or pharmacological inhibition or knockdown of glutaminase 1 (GLS1), the rate-limiting enzyme in glutaminolysis, impairs EC proliferation, while the role of glutamine in EC migration remains controversial (Huang et al., 2017; Kim B. et al., 2017). Interestingly, supplementation of asparagine in glutamine-depleted conditions restores protein synthesis and EC function (Huang et al., 2017; Pavlova et al., 2018). Moreover, reducing glutamate-dependent asparagine synthesis by silencing asparagine synthase limits EC sprouting (Huang et al., 2017).

Arginine can be converted by eNOS to citrulline and nitric oxide (NO), an endogenous gaseous signaling molecule that has a wide variety of biological properties that maintain vascular homeostasis and are atheroprotective, such as suppression of thrombosis, inflammation and oxidative stress (Tousoulis et al., 2012).

Valine metabolism generates 3-hydroisobutyrate (3-HIB) which promotes transendothelial FA transport and skeletal muscle FA uptake and storage, however how valine and 3-HIB affect EC metabolism and function remains to be determined (Jang et al., 2016).

Furthermore, *in vitro* and *in vivo* restriction of sulfur AAs methionine and cysteine triggers an angiogenic response by promoting endothelial VEGF and hydrogen sulfide production thereby shifting EC metabolism from oxidative metabolism to glycolysis (Longchamp et al., 2018).

## Endothelial cell metabolism in atherosclerosis

### Endothelial cell activation by the atherosclerotic microenvironment

**Figure 2:**
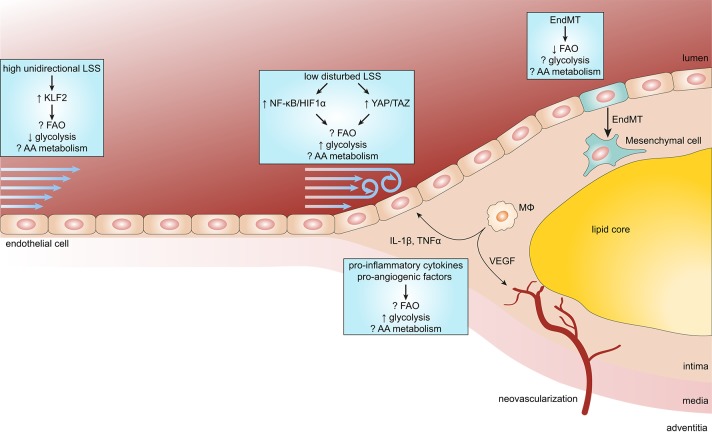
Endothelial metabolism in atherosclerosis. Endothelial cells (ECs) exposed to high unidirectional laminar shear stress (LSS) activate atheroprotective signaling via the transcription factor Krüppel-like factor 2 (KLF2) which reduces glycolysis and maintains ECs in a quiescent state. In contrast, atheroprone regions are subjected to low disturbed LSS enhances EC glycolysis via the nuclear factor κB (NF-κB)/hypoxia inducible factor 1α (HIF1α) axis and mechanotransducers Yes-associated protein (YAP) and transcriptional coactivator with PDZ-binding motif (TAZ). During the process of atherosclerosis, ECs are exposed to a pro-inflammatory milieu [e.g., cytokines interleukin 1β (IL-1β) and tumor necrosis factor α (TNFα)], which also enhances glycolysis in ECs. In more progressed lesions, macrophages (MΦ) start to secrete pro-angiogenic factors resulting in intraplaque neovascularization. Pro-angiogenic factors, like vascular endothelial growth factor (VEGF), enhance glycolysis in ECs to support proliferation and migration. In addition, reduced FAO in ECs predisposes them to undergo endothelial-to-mesenchymal transition (EndMT), which may affect plaque stability.

ECs remain mostly quiescent throughout adult life, however, they can become activated in response to various physiological and pathological stimuli (Gimbrone and García-Cardeña, 2016; Figure 2). Disturbed blood flow dynamics are an important initiating factor of EC activation preceding atherogenesis (Hajra et al., 2000). High unidirectional laminar shear stress (LSS) activates an atheroprotective gene expression program in ECs, including the upregulation of transcription factor Krüppel-like factor 2 (KLF2; Dekker et al., 2002). KLF2 regulates a network of genes that maintain vascular barrier integrity and confer EC quiescence, resulting in an anti-inflammatory, anti-thrombotic EC phenotype (Dekker et al., 2006). Interestingly, high LSS suppresses EC glucose uptake, glycolysis and mitochondrial respiration via a KLF2-dependent mechanism (Doddaballapur et al., 2015).

In contrast, ECs at atheroprone areas of the vasculature are subject to disturbed low LSS and show activation of pro-inflammatory pathways and enhanced expression of glycolytic enzymes (Feng et al., 2017). Low LSS enhances EC glycolysis via a nuclear factor κB (NF-κB)-hypoxia inducible factor 1α–(HIF1α)-dependent axis, despite being in a highly oxygenated environment (Feng et al., 2017; Wu et al., 2017). Moreover, pro-inflammatory cytokines increase glucose uptake and glycolysis in ECs which augments cytokine-induced NF-κB activation, most likely via lactate, however this requires further investigation (Folco et al., 2011; Végran et al., 2011; Cantelmo et al., 2016). Disturbed LSS and pro-inflammatory cytokines also activate the mechanotransducers Yes-associated protein (YAP) and transcriptional coactivator with PDZ-binding motif (TAZ), which promote a pro-inflammatory EC phenotype and atherosclerosis development (Wang K. C. et al., 2016; Wang L. et al., 2016b). Similar to NF-κB, YAP/TAZ signaling promotes EC glycolysis and vice versa (Enzo et al., 2015; Bertero et al., 2016; Kim J. et al., 2017). Partially blocking glycolysis by pharmacological inhibition of PFKFB3 reduces cancer cell adhesion to ECs and improves endothelial barrier function (Cantelmo et al., 2016), which may also limit leukocyte extravasation. Together, pro-inflammatory signaling enhances glycolysis and in turn glycolysis can drive pro-inflammatory programs, thereby forming a vicious cycle resulting in sustained pro-inflammatory signaling in ECs.

Furthermore, rupture-prone human atherosclerotic lesions are characterized by the presence of intraplaque neovascularization and hemorrhage (Virmani et al., 2005). Intraplaque Hypoxia and hemoglobin:haptoglobin complexes activate HIF1α-dependent signaling in macrophages leading to enhanced VEGF secretion, which in turn increases intraplaque angiogenesis, vascular permeability and leukocyte recruitment (Sluimer et al., 2008; Guo et al., 2018).

In contrast to glycolysis, the role of FAO in EC pro-inflammatory responses is not known. On the other hand, FAO maintains EC barrier function (Patella et al., 2015; Xiong et al., 2018). Furthermore, by undergoing EndMT, ECs contribute to the pool of mesenchymal cells within atherosclerotic lesions (Evrard et al., 2016). These EC-derived mesenchymal cells may contribute to plaque instability by enhanced expression and activity of matrix degrading proteins (Chen et al., 2015; Evrard et al., 2016). FAO has been shown to inhibit EndMT (Xiong et al., 2018), however, the role of glycolysis and AA metabolism herein remains to be determined. These studies suggest that endothelial FAO may reduce atherosclerosis development.

Besides its role in glycolysis, the pro-inflammatory YAP/TAZ pathway enhances EC glutaminolysis, suggesting a pro-atherogenic role for glutamine in ECs (Bertero et al., 2016). Interestingly, pharmacological inhibition of GLS1 does not affect pro-inflammatory gene expression in or leukocyte adhesion on ECs. Glutamine starvation, however, reduces protein synthesis resulting in ER stress which stimulates pro-inflammatory signaling and apoptosis (Tabas, 2010; Huang et al., 2017). In addition, glutamine deficiency reduces nucleotide synthesis and increased ROS production, resulting in reduced proliferation and increased apoptosis, respectively (Huang et al., 2017). Moreover, ECs have been shown to synthesize the neurotransmitter γ-aminobutyric acid from glutamate, which exerts anti-inflammatory effects in ECs (Sen et al., 2016). Therefore, it remains to be determined how EC glutaminolysis affects atherosclerosis development.

Interestingly, statins, the cholesterol-lowering drugs which are the cornerstone of atherosclerosis treatment, have been shown to increase KLF2 expression, while reducing pro-inflammatory signaling by NF-κB, HIF1α, and YAP/TAZ (Dichtl et al., 2003; Sen-Banerjee et al., 2005; Wang L. et al., 2016b), suggesting that statins may also exert their anti-atherogenic effects by reprogramming EC metabolism.

### Deregulated endothelial cell metabolism by risk factors for atherosclerosis

Risk factors, such as diabetes, obesity, and aging, have been shown to accelerate atherosclerosis development and are hallmarked by endothelial dysfunction and deregulated EC metabolism (Kanter et al., 2007; Wang and Bennett, 2012).

Diabetes is characterized by high glucose levels in the circulation which increases endothelial ROS production through auto-oxidation of glucose, NADPH-oxidases, eNOS uncoupling, and mitochondrial dysfunction, leading to DNA damage and subsequent activation of poly(ADP-ribose) polymerase 1 (PARP1) (Du et al., 2003; Forrester et al., 2018). ADP-ribosylation by PARP1 inhibits the glycolytic enzyme glyceraldehyde-3-phosphate dehydrogenase (GAPDH) resulting in the accumulation of glycolytic intermediates upstream of GAPDH and their redistribution to side branches of the glycolytic pathway, leading to (1) uncontrolled protein glycation via HBP, (2) production of advanced glycation end products (AGEs) through the polyol and methylglyoxal pathways, and (3) increased protein kinase C (PKC) activation through de novo synthesis of diacylglycerol from G3P (Du et al., 2003; Shah and Brownlee, 2016). Glycation of eNOS inhibits its activation, while PKC inhibits insulin-mediated activation of eNOS and increases the expression of the vasoconstrictor endothelin-1 (Du et al., 2001; Li et al., 2013). Furthermore, AGEs induce EC dysfunction through modification of proteins and ECM components, and activation of the receptor for AGEs resulting in activation of the pro-inflammatory NF-κB signaling pathway, increased vascular leakage and ROS production (Shah and Brownlee, 2016). Interestingly, reverting the glucose intermediates F6P and G3P toward the PPP by activating transketolase using a thiamine derivative reduces all three hyperglycemia-induced pathways described above as well as NF-κB activity (Hammes et al., 2003).

Obesity, as well as diabetes, is associated with elevated circulating concentrations of saturated FAs and triglyceride-rich lipoproteins, which can provide an additional supply of FAs (Goldberg and Bornfeldt, 2013; Nordestgaard, 2016). FAs can induce EC apoptosis and dysfunction by impairing NO-mediated vasodilation and promoting vascular permeability, oxidative and ER stress, pro-inflammatory NF-κB signaling and inflammasome activation (Inoguchi et al., 2000; Steinberg et al., 2000; Maloney et al., 2009; Tampakakis et al., 2016; Wang L. et al., 2016a). Reducing intracellular lipid levels by increasing FAO via peroxisome-proliferator-activated receptor (PPAR) β and δ-mediated upregulation of CPT1A or overexpression of PPAR-γ coactivator 1-α reduces FA-induced EC dysfunction and apoptosis, respectively (Won et al., 2010; Toral et al., 2015). Moreover, metformin, a first-line therapeutic drug against type 2 diabetes, decreases FA-induced ER stress and ROS production via a 5′ adenosine monophosphate–activated protein kinase-PPARδ-dependent axis (Cheang et al., 2014). However, why ECs do not increase FAO naturally upon lipid overload, despite having a considerable spare mitochondrial respiratory capacity, remains to be explored.

Cellular aging is a complex process characterized by the progressive loss of cellular function and is hallmarked, among others, by deregulated nutrient sensing and mitochondrial dysfunction (López-Otín et al., 2013). Indeed, ECs from aged rats are characterized by a reduction in mitochondrial mass, altered expression of mitochondrial components and an increase in mitochondrial ROS production (Ungvari et al., 2007, 2008). Furthermore, nutrient sensing pathways are fundamental to the aging process, since dietary restriction protects against the aging-mediated decline in EC function (Csiszar et al., 2014). Interestingly, endothelial-specific overexpression of the NAD^+^-dependent deacetylase sirtuin 1, a sensor that detects energy availability via NAD^+^, or supplementation of NAD precursors reverse the aging-associated decline in angiogenesis (Das et al., 2018).

Furthermore, additional hallmarks of aging, such as epigenetic alterations, and cellular senescence, may also affect EC metabolism. For instance, aging-induced epigenetic modifications can regulate the expression of metabolic genes and, conversely, metabolic intermediates modulate the epigenetic landscape (Brunet and Rando, 2017). Replicative senescence in ECs, achieved through consecutive *in vitro* passaging, reduced ATP levels by ~10-fold, despite having enhanced glycolysis, the main energy supplier in ECs (Unterluggauer et al., 2008; De Bock et al., 2013). Furthermore, inhibition of glutaminolysis induces a senescent phenotype in ECs *in vitro* (Unterluggauer et al., 2008). However, it remains to be determined which metabolic pathways are modulated by aging in ECs and whether EC metabolism can be targeted to reverse the aging-associated EC dysfunction.

## Concluding remarks

Metabolic pathways have emerged as key regulators of many EC functions, including angiogenesis, inflammation, and barrier function. However, despite major advances in our understanding in EC metabolism, there still remain many unanswered questions. Although research in endothelial AA metabolism is still in its infancy, limiting glycolysis and/or stimulating FAO in ECs may be a promising therapeutic strategy against atherosclerosis, even in the presence of risk factors such as diabetes and obesity. However, the majority of the complications due to atherosclerosis occur in the aged population (Shih et al., 2011). Therefore, a better understanding of the metabolic perturbations in aged ECs may provide additional therapeutic avenues in the treatment of atherosclerosis.

## Author contributions

All authors listed have made a substantial, direct and intellectual contribution to the work, and approved it for publication.

### Conflict of interest statement

The authors declare that the research was conducted in the absence of any commercial or financial relationships that could be construed as a potential conflict of interest.
